# Prognostic value of metabolic response in breast cancer patients receiving neoadjuvant chemotherapy

**DOI:** 10.1186/1471-2407-12-39

**Published:** 2012-01-25

**Authors:** Maria D Cao, Guro F Giskeødegård, Tone F Bathen, Beathe Sitter, Anna Bofin, Per E Lønning, Steinar Lundgren, Ingrid S Gribbestad

**Affiliations:** 1 Department of Circulation and Medical Imaging, Norwegian University of Science and Technology (NTNU), 7489 Trondheim, Norway; 2 St Olavs University Hospital, Trondheim, Norway; 3 Department of Laboratory Medicine, Children's and Women's Health, NTNU, 7489 Trondheim, Norway; 4 Department of Oncology, Haukeland University Hospital, 5021 Bergen, Norway; 5 Section of Oncology, Institute of medicine, University of Bergen, 5020 Bergen, Norway; 6 Department of Oncology, St. Olavs Hospital, University Hospital of Trondheim, 7006 Trondheim, Norway; 7 Department of Cancer Research and Molecular Medicine, NTNU, 7489 Trondheim, Norway; 8 Department of Circulation and Medical Imaging, The Faculty of Medicine, NTNU, MTFS, Postboks 8905, N-7489 Trondheim, Norway

## Abstract

**Background:**

Today's clinical diagnostic tools are insufficient for giving accurate prognosis to breast cancer patients. The aim of our study was to examine the tumor metabolic changes in patients with locally advanced breast cancer caused by neoadjuvant chemotherapy (NAC), relating these changes to clinical treatment response and long-term survival.

**Methods:**

Patients (n = 89) participating in a randomized open-label multicenter study were allocated to receive either NAC as epirubicin or paclitaxel monotherapy. Biopsies were excised pre- and post-treatment, and analyzed by high resolution magic angle spinning magnetic resonance spectroscopy (HR MAS MRS). The metabolite profiles were examined by paired and unpaired multivariate methods and findings of important metabolites were confirmed by spectral integration of the metabolite peaks.

**Results:**

All patients had a significant metabolic response to NAC, and pre- and post-treatment spectra could be discriminated with 87.9%/68.9% classification accuracy by paired/unpaired partial least squares discriminant analysis (PLS-DA) (*p *< 0.001). Similar metabolic responses were observed for the two chemotherapeutic agents. The metabolic responses were related to patient outcome. Non-survivors (< 5 years) had increased tumor levels of lactate (*p *= 0.004) after treatment, while survivors (≥ 5 years) experienced a decrease in the levels of glycine (*p *= 0.047) and choline-containing compounds (*p *≤ 0.013) and an increase in glucose (*p *= 0.002) levels. The metabolic responses were not related to clinical treatment response.

**Conclusions:**

The differences in tumor metabolic response to NAC were associated with breast cancer survival, but not to clinical response. Monitoring metabolic responses to NAC by HR MAS MRS may provide information about tumor biology related to individual prognosis.

## Background

The prognosis of patients with locally advanced breast cancer varies largely due to the heterogeneity of the disease, and 5-year survival rates from 50 to 80% have been reported [1]. Neoadjuvant chemotherapy (NAC) has been established as a standard treatment for locally advanced breast cancer, with anthracyclines and taxanes being among the most frequently used agents. NAC is provided to make primarily inoperable tumors resectable, and will also increase the rate of breast-conserving surgery without any significant increase in local or distal recurrence [2,3]. Studies investigating the metabolic responses and chemoresistance to single or a combination of drugs are important for effective treatment and better patient outcome.

Patients with a pathological complete response (pCR) after NAC have improved outcome compared to patients with residual disease, thus treatment response is a prognostic indicator. However, only ~20% of patients will achieve a pCR to NAC [4]. Other prognostic factors of breast cancer include axillary lymph node status, tumor size, Her-2 overexpression, histopathological grade, and hormone receptor status. The status of Her-2 and hormone receptors is also predictive of treatment response. Identification of other markers for prognosis and treatment response may help stratify patients for better individualized treatment.

Several studies have shown altered metabolism in cancer compared to normal tissue. Elevated levels of total choline-containing compounds (tCho) are frequently observed in cancer, and may serve as magnetic resonance spectroscopy (MRS) markers for malignancy, both *in vivo *and *ex vivo *[5,6]. The tCho signal constitutes signals from glycerophosphocholine (GPC), phosphocholine (PC) and free choline (Cho) which are involved in phospholipid metabolism through the Kennedy pathway. A decreased level of tCho detected by *in vivo *MRS has been suggested as a possible marker for treatment response [7,8]. Altered concentrations of other tissue metabolites, such as increased levels of lactate, have also been associated with malignancy [9,10]. Elevated lactate levels may be related to hypoxia, a common feature of solid tumors where glucose is catabolised to lactate due to the lack of oxygen. Also under conditions with sufficient oxygen levels, cancer cells may convert glucose to lactate, described as the Warburg effect.

High resolution magic angle spinning (HR MAS) MRS is a non-destructive technique providing highly resolved MR spectra of intact tissues with minimal sample preparation. HR MAS MR spectra provide an overview of the different metabolites that are present in a tissue sample, and can give insight into the complex processes leading to cancer and other diseases. More than 30 metabolites have been identified in breast tissue using HR MAS MRS [11]. Systematic studies of the metabolic state of biological systems using multivariate analysis methods are referred to as metabolomics. MR metabolomics studies of breast cancer have revealed correlations between tissue metabolic profiles and clinical prognostic factors such as hormone receptor status, grade and lymphatic spread [12-14]. Long-term survival of breast cancer patients has been successfully predicted from breast cancer tissue using multivariate classification models [15]. The purpose of this study was to examine the metabolic changes in breast cancer tissues resulting from treatment with NAC, and to relate these changes to treatment response and long-term survival. This is the first study to investigate the metabolic response of NAC in a large breast cancer cohort using *ex vivo *MRS.

## Methods

### Patient and tumor characteristics

We examined a subcohort of breast cancer patients (n = 89) from a larger open-label multicenter study where patients were randomly allocated to receive NAC treatment with either anthracycline (epirubicin, 90 mg/m^2^) or taxane (paclitaxel, 200 mg/m^2^) monotherapy [16]. The patients were given subsequent adjuvant endocrine treatment according to guidelines from the Norwegian Breast Cancer Group. The inclusion criteria and treatment protocol are fully described elsewhere [16]. Briefly, female breast cancer patients at pre/post menopausal age (≤ 70 years) with locally advanced (stage III, T_3/4 _and/or N_2_) non-inflammatory breast cancer with or without limited distant metastasis were recruited in the period 1997-2003. The patients were treated every third week for four cycles. Patients showing a non-satisfactory response were assigned to the opposite treatment. From each patient, an incisional biopsy was taken before treatment with NAC and a post-treatment biopsy was excised during surgical removal of the tumor. The biopsies were immediately snap-frozen and stored in -80°C and subsequently in liquid nitrogen in a biobank until use. A part of the pre-treatment tumor biopsy was obtained for routine pathological diagnosis and hormone status assignment. Estrogen (ER) and progesterone receptor (PgR) status were determined by immunohistochemical staining (positive ≥ 10% staining cells). The study was approved by The Regional Committee for Medical and Health Research Ethics (Norwegian Health Region III) and informed written consent was obtained from all patients

### Response and survival evaluation

Response to treatment was evaluated using the WHO criteria by the UICC system [17]. Treatment response was assessed clinically by comparing caliper measurements prior to NAC treatment and after the last cycle. In the subcohort included in this study, the patients were classified to have either partial response (≥ 50% reduction in tumor size (the product of the two largest tumor diameters) after treatment, but not complete response) or stable disease (< 50% reduction to ≤ 25% increase in tumor size after treatment). Patients deceased within 5 years after diagnosis were classified as non-survivors whereas patients surviving 5 years or more were classified as survivors.

### Histopathological examinations

Prior to HR MAS MRS analysis, imprint cytology smears were prepared from the tissue samples and stained with the May-Grünwald-Giemsa stain (Color-Rapid, Med-Kjemi, Norway). Confirmation of tumor cell content was determined microscopically by a cytopathologist.

### HR MAS MRS experiments

HR MAS MRS analyses were performed on a Bruker Avance DRX600 spectrometer (Bruker Biospin GmbH, Germany) equipped with a ^1^H/^13 ^C MAS probe with gradient. The run order of the samples was randomized http://www.random.org and blindly analyzed during 18 days. Each sample (15.1 ± 2.8 mg) was cut to fit a 30 μl leak-proof disposable insert (Bruker Biospin Corp, USA) and added phosphate buffered saline (PBS, 3 μl) in D_2_O containing trimethylsilyl tetradeuteropropionic acid (TSP, 98.2 mM) for chemical shift referencing. Samples were spun at 5 kHz and spectra were recorded within 31 min per sample at 4°C to minimize tissue degradation. Spin-echo spectra (cpmgpr; Bruker) were recorded as previously described [14].

### Data preprocessing

Twenty eight spectra were excluded from further studies due to low tumor cell content. The resulting data set consisted of 150 spectra from 85 patients (70 pre-treatment and 80 post-treatment spectra). Characteristics of the included patients and tumors are listed in Table 1. The MR spectra were Fourier transformed into 128 K after 0.3 Hz exponential line broadening. Chemical shifts were referenced to the TSP peak at 0 ppm. The spectral region between 4.69 and 1.45 ppm, excluding the water peak and large lipid residuals, was chosen for analysis. Signals from ethanol pollutions between 3.69 and 3.57 ppm were removed together with lipid residual signals between 3.01 and 1.52 ppm. The spectra were baseline corrected using asymmetric least squares [18] with parameters λ = 1e7 and *p *= 0.0001, and the minimum value of each spectrum was set to zero by subtracting the lowest value. The spectra were normalized to equal total area, and peak aligned using icoshift [19].

**Table 1:** Patient and tumor characteristics

		**Survivors**	**Non-survivors**	NA*
		**(n = 60)**	**(n = 23)**	**(n = 2)**
Mean age (± SD)	years	51.1 ± 10.6	49.3 ± 8.3	46.4 ± 2.6
Mean tumor dimensions (mean ± SD)	mm	67.9 × 67.9 ± 18.0 × 19.8	78.6 × 77.3 ± 22.0 × 24.0	65.0 × 51.5 ± 7.1 × 26.2
NAC treatment	Epirubicin	25	8	-
	Paclitaxel	23	5	2
	Both^1^	12	10	-
Treatment	Partial response	40	11	1
response	Stable disease	20	12	1
AJCC	IIB	21	7	1
	IIIA	25	11	1
	IIIB	12	2	-
	IV	2	3	-
ER status	+	41	7	2
	-	19	15	-
	unknown	-	1	-
PgR status	+	32	14	2
	-	28	18	-
	unknown	-	1	-
Nodes	+	32	14	1
	-	28	9	1
Metastasis	+	2	2	0
	-	58	21	2

*NA, not applicable--One patient without following up and one patient dead by other causes; AJCC, American Joint Committee on Cancer; Both^1^, sequential treatment with Epirubicin and Paclitaxel

### Multivariate data analysis

Partial least squares (PLS) analysis is a regression method for analysis of collinear data with numerous variables. The method is based on extraction of underlying structures, or latent variables (LVs), that maximize the covariance between X (the spectra) and a response variable Y [20]. PLS discriminant analysis (PLS-DA) attempts to discriminate between distinct classes. PLS-DA was performed in Matlab R2009a (The Mathworks, Inc., USA) using PLS_Toolbox 6.2.1 (Eigenvector Research, USA). A PLS-DA model was built on mean-centred spectra from randomly chosen training samples (90% of the patients) and used to predict the status of test samples (the remaining 10%). This procedure was repeated 20 times and the average classification results were calculated. The number of LVs to use was chosen by cross-validation of the whole data set and used for all repetitions to avoid biased results. The importance of each variable in the loadings of the PLS-DA was evaluated by variable importance in the projection (VIP) scores [21]. The VIP score positively reflects the variable's influence on the classification, and variables with a score greater than one are generally considered important [21,22]. To evaluate the statistical significance of the classification results, permutation testing was performed [23]. In permutation testing, the class labels are permuted to resemble random classification. It is then possible to examine if the achieved prediction results of the original data set are significantly different than random predictions. The data set with the permuted class label was divided into training and test sets repeated 20 times as described for the original data set, and the average results were calculated. The permutation procedure was repeated 1,000 times, and the prediction error of the original data set was compared to the distribution of prediction errors from the permutation. P-values ≤ 0.05 were considered significant.

Multilevel PLS-DA [24,25] is an extension of ordinary PLS-DA which can be used as a paired analysis for multivariate data. This analysis can only be used when the data has a multilevel structure, i.e. when interventions are evaluated on the same subject. In multilevel PLS-DA, the *between *subject variation is separated from the *within *subject variation. This is useful in metabolic profiling as the variation between subjects, resulting from differences in age, disease state, genetics and other factors, can obscure the metabolic changes caused by the intervention. The between subject variation is described by the average of the two observations (pre- and post-treatment) from each subject, whereas the within subject variation is described by the net difference between the two observations (pre- and post-treatment). Multilevel PLS-DA was used to examine metabolic changes in the spectra resulting from NAC treatment. The split-up of variation was done using algorithms made available by van Velzen et al. [24]. Further PLS-DA classifications of the within subject variation were performed using PLS_Toolbox as described for the unpaired analyses. The net difference of the spectra pre- minus post-treatment (positively representing the metabolites higher expressed before treatment) is annotated as control, while the net difference post- minus pre-treatment (positively representing the metabolites higher expressed after treatment) is annotated as treatment. More specifically,

(1) $$\begin{array}{c} \text{control }=A-B\\ \text{treatment }=B-A \end{array}$$

where the matrix **A **represents pre-treatment spectra, and matrix **B **represents the post-treatment spectra.

### Univariate data analysis

To further validate the important metabolites from the PLS-DA models, relative intensities were found by integrating the peak areas of spectra normalized to equal total areas after removal of lipid residuals (Matlab R2009a, The Mathworks, Inc., USA). Normalization of spectra with the lipid residual signals removed will correct for differences in sample size and tumor cell content, as it can be assumed that most of the lipid signals from breast samples do not originate from cancer cells. Group differences were statistically tested by Wilcoxon rank sum tests or Wilcoxon sign rank for paired analyses, and considered significant if the p-values were ≤ 0.05.

## Results

### Metabolic response to neoadjuvant chemotherapy

**Figure 1:**
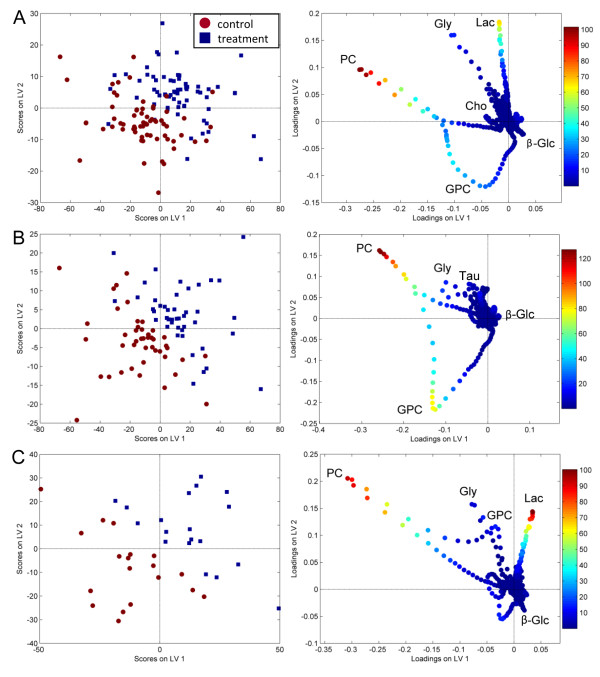
**Scores and loadings from multilevel PLS-DA**. The score and loading plots show the two first latent variables separating treatment and control spectra based on (A) the whole data set, (B) 5-year survivors, and (C) non-survivors. The variables in the loadings are colored according to VIP scores, indicating the importance of each variable in the discrimination. The control spectra equal the difference between pre- and post-treatment spectra, while the treatment spectra equal the difference between post- and pre-treatment spectra. The most protruding differences in the loading plots of (B) survivors and (C) non-survivors show a decrease in GPC levels in survivors after treatment, while non-survivors have increased levels of lactate. PC appears to be present in high levels in some samples of both survivors and non-survivors. Lac, lactate; Gly, glycine; β-Glc, β-glucose.

All classification results are summarized in Table 2. An unpaired PLS-DA of the pre- and post-treatment spectra of the whole data set showed a significant difference in the metabolite profiles in response to NAC treatment, indicating a metabolic response to NAC in all patients. However, the specificity of the classification was low (57.1%). When comparing the classification errors of PLS-DA and paired multilevel PLS-DA from 20 different test sets, the multilevel PLS-DA with split-up of the variation decreased the classification error significantly (Wilcoxon rank sum test, *p *< 0.001), showing the beneficial effect of the paired analysis. Treatment and control spectra could be separated with a sensitivity and specificity of 87.9%. Figure 1A shows the scores and loadings of the multilevel PLS-DA. Lactate and PC were of high importance for the discrimination according to the VIP scores in the loadings. The levels of lactate and glycine appear to be increased in response to treatment, while the levels of PC are markedly decreased for some patients. In addition, GPC levels appear to be decreased in response to treatment. No clustering according to the given chemotherapeutic agents could be seen in the multilevel PLS-DA score plot (results not shown), thus the metabolic treatment effects of epirubicin and paclitaxel appear to be indistinguishable.

**Table 2:** Classification results from multilevel PLS-DA and PLS-DA

				Class. model	No. of LVs	Variance X/Y (%)	Sensitivity/ Specificity (%)	Class. accuracy (%)	Permutation p-value
Paired data	All samples	Control vs Treatment	(n = 65p)	Multilevel PLS-DA	2	58.9/50.9	87.9/87.9	87.9	< 0.001
	Partial response	Control vs Treatment	(n = 37p)	Multilevel PLS-DA	2	55.7/61.7	80.0/80.0	80.0	< 0.001
	Stable disease	Control vs Treatment	(n = 28p)	Multilevel PLS-DA	2	63.2/61.3	88.7/88.7	88.7	< 0.001
	Survivors	Control vs Treatment	(n = 44p)	Multilevel PLS-DA	2	60.0/50.5	83.0/83.0	83.0	< 0.001
	Non-survivors	Control vs Treatment	(n = 19p)	Multilevel PLS-DA	2	70.6/63.9	82.5/82.5	82.5	0.006

Unpaired data	All samples	Pre- vs Post-treatment	(n = 65p)	PLD-DA	2	50.3/29.2	80.7/57.1	68.9	< 0.001

	Pre-treatment	Partial response vs Stable disease	(n = 48) (n = 32)	PLS-DA	2	51.5/19.3	56.7/60.2	58.4	0.231
	Post-treatment	Partial response vs Stable disease	(n = 41) (n = 29)	PLS-DA	NaN	-	-	-	-
	Pre-treatment	Survivors vs Non-survivors	(n = 57) (n = 20)	PLS-DA	NaN	-	-	-	-
	Post-treatment	Survivors vs Non-survivors	(n = 47) (n = 21)	PLS-DA	3	62.8/32.1	58.4/75.3	70.1	0.009
	Post-treatment	Epirubicin vs Paclitaxel	(n = 29) (n = 23)	PLS-DA	2	45.7/26.8	45.0/48.3	46.5	0.245

The sensitivity is for detecting a treatment/stable disease/non-survivor/Paclitaxel spectrum; Variance X/Y, amount of variance from X/Y explained by the model; NaN, no valid model; p, pairs

### No differences in metabolic response between clinical response groups

The patients were divided into two groups according to their clinical response (partial response or stable disease), and multilevel PLS-DA was performed on each group separately in order to discover potential differences in metabolic treatment response between the groups. Both for patients with partial response and stable disease there was a significant change in the tumor metabolism in response to NAC treatment, and treatment spectra could be discriminated from controls with a sensitivity and specificity ≥ 80.0%. The metabolic response to NAC as observed in the loading plots was similar for both subgroups, resembling the changes observed for the whole data set (results not shown). Thus, no difference in the metabolic response could be detected between patients with stable disease and partial response.

### Different metabolic responses correlate with survival

The patients were also divided into two groups according to their survival status (5-year survivors or non-survivors). Both for survivors and non-survivors there was a clear change in the tumor metabolism in response to NAC treatment (Figure 1B and 1C), and treatment spectra could be discriminated from controls with a sensitivity and specificity ≥ 82.5%. However, the metabolic treatment response appears to differ between survivors and non-survivors.

The loadings showed unchanged lactate levels in response to treatment in survivors, while lactate increased in non-survivors with high importance for the discrimination according to the VIP scores. This was confirmed by comparison of the relative intensities from metabolite integrals, showing a significant increase in lactate levels in response to treatment in non-survivors (*p *= 0.004) but not in survivors (Table 3).

**Table 3:** Changes in relative intensities of metabolites in response to NAC

		Survivors (n = 44)		Non-survivors (n = 19)	
		
Metabolite	ppm	Mean ± SE	p-value	Mean ± SE	p-value
Lactate	4.08-4.13	2.8 ± 15.3	0.815	97.1 ± 26.4	0.004**
Glycine	3.54-3.56	-19.6 ± 8.0	0.047*	0.8 ± 14.3	0.601
GPC	3.22-3.24	-59.6 ± 14.6	< 0.001**	-11.8 ± 15.7	0.469
PC	3.21-3.22	-95.4 ± 24.3	< 0.001**	-67.4 ± 44.2	0.227
Cho	3.20-3.21	-16.6 ± 6.3	0.013*	-17.8 ± 6.3	0.084
tCho	3.20-3.24	-167.2 ± 36.7	< 0.001**	-95.3 ± 57.5	0.091
Taurine	3.40-3.43	-0.8 ± 13.2	0.861	6.9 ± 12.5	0.398
β-Glucose	4.61-4.64	17.8 ± 5.1	0.002**	-0.2 ± 8.8	0.841

The values (post- minus pre-treatment) of relative intensities are integrated peak areas from spectra normalized to equal total areas. Wilcoxon sign rank tests were used for paired statistical analyses. *p < 0.05, ** *p *< 0.01

Glycine appears to be decreased in survivors according to the loadings, and the difference in relative intensities before and after treatment was significant (*p *= 0.047). For non-survivors, the glycine level appears to be high in some samples from both the control and the treatment group; hence the role of glycine in the loading plot is more difficult to interpret. The glycine change from integrated relative intensities was not significant in non-survivors, with a mean value close to zero.

GPC levels were decreased in the loading plot of survivors with VIP scores showing high importance, while changes in GPC levels in non-survivors were less important for the discrimination. Accordingly, the relative intensities of GPC were significantly lower in response to treatment in survivors (*p *< 0.001) but not in non-survivors.

The loadings show decreased levels of PC in response to treatment in both survivors and non-survivors. However, the change in PC relative intensities was only significant for survivors (*p *< 0.001), but not for non-survivors, possibly due to a high standard error.

Relative intensities of Cho levels were significantly decreased in survivors (*p *= 0.013) in response to treatment, but only a trend of decreased Cho levels was seen in non-survivors (*p *= 0.084). In addition, glucose was significantly increased in survivors (*p *= 0.002). Cho and glucose were not protruding in the loadings, possibly due to low intensity values.

As an overall measure of the partly overlapping choline-containing metabolite peaks (GPC, PC, and Cho), the changes in relative intensities of tCho were calculated. Survivors had a significant decrease in tCho levels in response to treatment (*p *< 0.001), while a trend of decreased tCho levels were detected in non-survivors (*p *= 0.091).

### Metabolic traits at pre- and post-treatment

**Figure 2:**
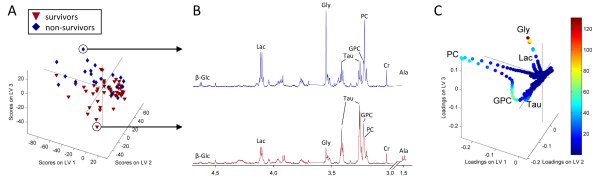
**PLS-DA of the MR spectra from biopsies excised post-treatment**. (A) A score plot separating survivors and non-survivors, (B) Representative spectra showing the metabolic differences of the tumors of survivors and non-survivors. (C) The loadings of the PLS-DA model with variables colored according to the VIP scores. β-Glc, β-glucose; Lac, lactate, Gly, glycine; Cr, creatine; Ala, alanine.

A PLS-DA of the post-treatment spectra showed a significant difference in the metabolite profiles of 5-year survivors and non-survivors after treatment with 70.1% correct classification (Table 2). According to the scores and loadings shown in Figure 2, the tumors of non-survivors appear to have more of the metabolites lactate and glycine, and less GPC and taurine than survivors post-treatment. PC appears to be present in high levels in some samples of both survivors and non-survivors. The glycine level was denoted to be of major importance according to the VIP scores, and the relative intensities of glycine were significantly higher in non-survivors compared to survivors post-treatment (*p *= 0.033, Table 4). Similarly, a trend of higher relative intensities of lactate was observed in non-survivors (*p *= 0.089). No significant differences in the relative intensities of taurine and GPC were observed, however differences in the levels of tCho approached significance (*p *= 0.075) with non-survivors having higher relative intensities than survivors post-treatment.

**Table 4:** Relative intensities of metabolites at pre- and post-treatment

		Pre-treatment (mean ± SE)			Post-treatment (mean ± SE)		
		
Metabolites	ppm	Survivors	Non-survivors	p-value	Survivors	Non-survivors	p-value
Lactate	4.08-4.13	185.8 ± 11.4	164.6 ± 13.8	0.534	196.2 ± 12.7	250.6 ± 26.0	0.089
Glycine	3.54-3.56	115.0 ± 6.4	120.8 ± 11.0	0.542	91.2 ± 4.1	111.4 ± 8.4	0.033*
GPC	3.22-3.24	167.3 ± 12.4	170.1 ± 23.9	0.949	115.6 ± 6.7	153.9 ± 24.1	0.144
PC	3.21-3.22	247.9 ± 17.0	285.4 ± 34.9	0.338	158.0 ± 16.1	205.5 ± 28.1	0.105
Cho	3.20-3.21	95.4 ± 3.9	108.7 ± 8.3	0.225	79.5 ± 3.7	85.9 ± 6.2	0.276
tCho	3.20-3.24	498.3 ± 26.4	551.3 ± 46.8	0.253	344.9 ± 22.3	434.6 ± 44.6	0.075
Taurine	3.40-3.43	242.6 ± 9.6	222.8 ± 12.4	0.393	248.0 ± 8.1	222.3 ± 9.3	0.144
β-Glucose	4.61-4.64	48.4 ± 3.3	49.3 ± 6.0	1.000	63.6 ± 4.3	53.5 ± 6.6	0.172

The values of relative intensities are integrated peak areas from spectra normalized to equal total areas. Wilcoxon rank sum tests were used for statistical analyses. *p < 0.05, ** *p *< 0.01

The metabolic differences between survivor and non-survivors were not seen pre-treatment as the multivariate model could not discriminate the two outcome groups (model not valid). Furthermore, none of the metabolites showed significant differences in relative intensities between survivors and non-survivors pre-treatment (Table 4).

No significant differences in the metabolite profiles at pre- or post-treatment were detected between patients with partial responders and stable disease by PLS-DA. Post-treatment spectra from patients treated with Epirubicin and Paclitaxel could not be discriminated by PLS-DA, further confirming the similarity of the metabolic response of the two chemotherapeutic agents that were used in this study.

## Discussion

In this study we examined the metabolic effect of NAC treatment in patients with locally advanced breast cancer. By comparing MR spectra of biopsies taken pre- and post-treatment, significant metabolic changes in response to treatment were found both by paired and unpaired multivariate models. The results using paired multilevel PLS-DA were however superior to those of unpaired PLS-DA, thus taking advantage of the multilevel structure in the data set was clearly beneficial.

Epirubicin and Paclitaxel appear to affect the metabolism of the tumor cells in the same manners, as evidenced both by indistinguishable metabolic responses and similar metabolic traits of the post-treatment spectra. Anthracyclines work by inducing damage to the DNA, while taxanes stabilize the microtubules; thereby inhibiting cell division [26,27]. However, both treatments will eventually result in cell death. This might explain why the two agents appear to have similar metabolic responses.

Interestingly, all patients in our study cohort experienced changes in the metabolite profiles in response to treatment, including also patients categorized to have a clinically stable disease. No differences in the metabolic responses of the clinical response groups were detected. However, when examining the metabolic changes in survivors and non-survivors independently, a difference in the metabolic response to NAC was seen. Non-survivors had a significant increase in lactate levels in response to treatment, while survivors showed no change in lactate levels. As a result, a trend of higher levels of lactate was detected in non-survivors compared to survivors post-treatment. Increased lactate levels may be a marker for tumor aggressiveness as high levels of lactate have been correlated with low survival rates, high incidence of distant metastasis and recurrence, and increased risk of radiation resistance in several types of cancer [28-30]. Modification of cell energy metabolism is typically observed in malignant tumors and is suggested as an emerging hallmark of cancer [31]. Under normoxic conditions, cancer cells can reprogram their energy metabolism to largely depend on aerobic glycolysis as their primary energy pathway resulting in increased lactate production; the so-called Warburg effect. It is not fully known why cancer cells prefer aerobic glycolysis over complete oxidation as this would produce far more ATP. It has been hypothesized that lactate may enhance the invasiveness of tumor cells and the resulting low pH may help tumor cells evade tumor-attacking immune cells [32].

In addition to aerobic glycolysis, breast cancer cells are often hypoxic due to poor blood supply [33]. It can be assumed that the large tumors of patients with locally advanced breast cancer will be affected by hypoxia. Hypoxia can induce the transcription factor hypoxia inducible factor-1α (HiF-1α), which in turn upregulates multiple genes involved in the glycolytic pathway, angiogenesis, cell proliferation, and other mechanisms [33-35]. Furthermore HIF-1α promotes transcription of lactate dehydrogenase (LDH) and lactate monocarboxylate transporters (MCT), and thus plays an important role in the production and efflux of lactate in cancer cells [36,37]. Inhibition of LDH by small interfering RNA (siRNA) in mouse breast tumors has been shown to reduce the glycolytic activity associated with a decrease in tumor proliferation and tumorigenic potential [38]. Thus we can suggest that the increased levels of lactate after NAC treatment observed in non-survivors may reflect enhancement of aerobic glycolytic activity and/or hypoxic tumor responses that confer higher tumor malignancy and poor prognosis. In addition, the glucose levels were increased in response to treatment in survivors but not in non-survivors. Studies using ^18^F- FDG PET have shown decreased glucose uptake in tumors after treatment with chemotherapy [39,40]. The increased glucose level observed in survivors after NAC may therefore be indicative of decreased aerobic glycolysis and tumor hypoxic response favorable of long term breast cancer survival.

Survivors had a significant decrease in glycine as a response to treatment, while it remained unchanged in non-survivors. This was reflected in the post-treatment spectra, showing significantly lower levels of glycine in survivors. In a previous study of patients receiving NAC with the anthracycline doxorubicin, we also found decreased glycine levels after NAC to be associated with long term breast cancer survival [15]. The biological role of glycine in tumor malignancy is still unclear. Several studies have elucidated the biomarker potential of glycine in human brain tumors, where it was found to positively correlate with tumor grade [41,42]. Higher levels of glycine have also been detected in pre-clinical studies of the more aggressive basal-like breast cancer model compared to the luminal-like model [43]. In patients, high glycine levels detected in malignant breast tumors have been correlated with poor prognosis [44]. Glycine is mainly synthesized from 3-phosphoglycerate, an intermediate of the glycolysis. In addition, glycine can be synthesized from Cho through the glycine-betaine pathway. We can postulate that the decreased glycine levels after NAC treatment detected in survivors are caused by altered glycolysis and/or reduced Cho levels associated with reduced tumor aggressiveness.

A significant decrease of GPC, PC, Cho levels and the combined tCho level was detected in survivors in response to treatment, whereas non-survivors experienced only a trend of decrease in Cho and tCho levels. As a result, lower tCho levels in survivors compared to non-survivors post-treatment approached significance. In a previous publication, we showed that GPC and Cho concentrations significantly decreased in patients with long-term survival (≥ 5 years), while non-survivors (< 5 years) had no significant changes in choline phospholipid metabolites in response to doxorubicin [15]. Choline phospholipid metabolites are important biological compounds in cell membrane synthesis and turnover. In addition, tCho levels have been associated with increased malignancy and activation of oncogenic signaling in breast cancer cells [45,46]. Higher tCho concentrations have been detected in high-grade breast tumors and tumors with higher pharmacokinetic parameters measured with dynamic contrast enhanced MR imaging, indicating a correlation between choline phospholipid metabolism and tumor malignancy and angiogenesis [47,48]. As previously mentioned, cancer cells may undergo adaptive responses to hypoxia by inducing HiF-1α. Increased tCho levels and choline kinase alpha (CHKA) expressions have been detected in prostate cancer cells and xenografts models under hypoxic compared to normoxic conditions [49]. In the same study, the authors found hypoxic tumor regions to be co-localized with regions of high tCho, which possibly occurred through the up-regulation of CHKA by HiF-1α. CHKA is known to play an important role in malignant transformation in several types of cancer [50]. Overexpression of CHKA and elevated PC and tCho levels of breast cancer cells have been associated with increased invasiveness and drug resistance [51]. Decreased choline phospholipid metabolism after NAC treatment may be associated with lower malignancy that potentially can be used as a predictor of breast cancer survival.

The metabolic responses to NAC treatment appear to be similar in patients with partial response and stable disease. None of the patients in this study had a progressive disease, whereas patients with a complete response would not have any tumor tissue left for a post-treatment biopsy. By definition the group with stable disease can have up to 50% reduction in tumor volume, and indeed only two patients in this study had an equal or increased tumor size after NAC. In that respect, almost all patients had a biological effect of the treatment although the tumor reduction was small for patients with a stable disease. It is conceivable that a cohort including also patients with progressive disease would reveal clearer differences in metabolic response between the clinical response groups. It is however noteworthy that all patients in this study in general had a decrease in tCho after NAC, as tCho is suggested as an *in vivo *biomarker for clinical treatment response. Using current *in vivo *systems, the different choline-containing compounds can not be separated by ^1^H MRS, and metabolites such as lactate and glycine are not visible using standard *in vivo *protocols. *Ex vivo *studies can therefore provide information that is not achievable in current clinical *in vivo *systems. However, future technological improvements of *in vivo *systems and the implementation of spectral editing and hyperpolarization techniques can enable the translation of *ex vivo *findings into *in vivo *clinical use.

In this patient cohort, the prediction of overall survival was accomplished with 70.1% classification accuracy using post-treatment spectra, but no prognostic information could be extracted from the pre-treatment spectra. This shows that the difference between survivors and non-survivors post-treatment results from a metabolic response to the treatment. The observed higher levels of lactate and glycine in non-survivors compared to survivors support our previous studies postulating high lactate and glycine levels to be predictive of low breast cancer survival rates (< 5 years).

The tissue samples examined in this study have been stored in liquid nitrogen since 1997-2003. However, no significant metabolite degradation due to storage time has been reported using HR MAS MRS [52]. In addition, the tissue samples were prepared on ice and MR experiments were performed at 4°C which will reduce the degradation process. The metabolic patterns observed in this study should therefore not be affected by sample storage and preparation.

Prediction of survival in patients receiving NAC is challenging. As NAC will downstage and potentially completely remove the disease, standard prognostic indicators such as tumor size and lymph node status are no longer fully applicable after NAC. Several studies have shown that a pathological complete response after NAC is associated with better survival rates [4]. However, approximately 80% of patients will have residual tumor in the breast after treatment [4]. Our study shows that the metabolic response to treatment may be an indicator of patient prognosis.

## Conclusions

By comparing HR MAS MR spectra from biopsies excised before and after NAC treatment, we have revealed significant metabolic changes in breast cancer tumors as a response to treatment. Different metabolic responses could be related to patient outcome, but did not separate patients with partial response from those with stable disease after treatment. Non-survivors had increased tumor levels of lactate after treatment, while survivors experienced a decrease in the levels of glycine and choline-containing compounds. These differences in tumor response may reflect tumor aggressiveness associated with breast cancer survival. Monitoring metabolic responses to NAC by HR MAS MRS may provide information about tumor biology related to prognosis, and help identify pathways for targeted therapies.

## Competing interests

The authors declare that they have no competing interests.

## Authors' contributions

MCD carried out the HR MAS MRS and imprint cytology experiments. GFG carried out Matlab programming and preprocessing of the spectral data. MDC and GFG performed the statistical analyses, interpretations of the results and writing of article. ISG, TFB, and BS participated in the design of the study, interpretations of the results and writing of article. AB analyzed the imprint cytology samples and helped to draft the manuscript. PEL and SL recruited the patients, collected the tumor biopsies and helped to draft the manuscript. All authors read and approved the final manuscript.

## Pre-publication history

The pre-publication history for this paper can be accessed here:

http://www.biomedcentral.com/1471-2407/12/39/prepub
